# *In vitro* Evaluation of the Accuracy of the Root Zx in the Presence of Naocl 2.5% and Chlorhexidine 0.2%

**DOI:** 10.4317/jced.54193

**Published:** 2018-11-01

**Authors:** Marjan Bolbolian, Siamak Golchin, Seyedmatin Faegh

**Affiliations:** 1 Assistant Professor of Endodontics, Dental Caries Prevention Research Center, Qazvin University of Medical Science, Qazvin, Iran; 2 General Dentist, Student Research Committee, Qazvin University of Medical Sciences, Qazvin, Iran; 3 Dental Student, Faculty of Dentistry, Semmelweis University, Budapest, Hungary

## Abstract

**Background:**

Establishing the working length at the apical constriction is considered ideal for root canal therapy. Because of the limitations of radiography and complicacy of the apex of the root, electronic measurement of canal length has become valuable for endodontic treatment. This study was designed to evaluation of the accuracy of the Root zx electronic apex locator in the presence of NaOCl 2.5% and chlorhexidine 0.2%.

**Material and Methods:**

Thirty extracted human premolars with complete root formation were enrolled. The actual length (AL) was assessed visually (under stereo microscope) and teeth mounted in the saline model. The electronic length (EL) measurements were recorded in the presence of NaOCl 2.5% and chlorhexidine 0.2% and the differences between the EL and AL were compared.

**Results:**

By accepting the error of 0.5 and 1 mm, the accuracy of Root zx was 76.7% and 96.7% in the presence of chlorhexidine 0.2% and 90% and 100% in the presence of NaOCl 2.5%, respectively. No statistical differences was found between the measured groups (*P*=0.223).

**Conclusions:**

Our results confirmed that Root zx can accurately determine the apical constriction in presence of both NaOCl 2.5% and chlorhexidine 0.2%.

** Key words:**Chlorhexidine, Sodium Hypochlorite, Root ZX.

## Introduction

Exact determination of the canal length is the prerequisite of successful root canal treatment and decreases the chance of incomplete cleaning and damages to periapical tissue due to over-preparation more than working length. The common methods for determination of working length include radiography, knowing the average teeth length, tactile sensation, and moisture on the paper cone which all of them have certain limitations (1). Determination of working length just by X-ray had some problems such as image distortion, shortening or elongation of the length, different interoperation, and lack of 3-dimensional image. Also, X-ray image just has 5% magnification. Achieving of working length by subtraction of 1 mm from radiographic apex can be provide shorter or longer working length in comparison to exact working length. Therefore, use of this rule as a general rule is untrustworthy (2).

Construction and development of different generations of apex locator is considerably helped to more accurate and predictable determination of working length. Because using of these apparatuses, the place of apical constriction, which is the most appropriate place for ending of root treatment, is determined. Albeit, it must be noted that apical constriction has high morphological variations and exact determination of that is not possible (3).

Root zx apex locator (J. Morita Inc., Tokyo, Japan) is the third generation of apex locator which don’t need calibration and its microprocessor calculated the impedance ration. The third generation apparatuses use several frequencies for determination of end of canal. Indeed, the basis of Root zx apex locator is changes of electrical capacity at the apical constriction (4).

Based on different studies, accuracy of Root zx apex locator is varied in the presence of different electrolytes which used for rinsing or opening the canal (3,5) and reported as 82.3%-96.2% (by accepting ± 0.5 error) (3). Based on the claim by manufactures about the appropriate function of Root zx in the presence of different irrigants and needs no calibration, we aimed to evaluate the accuracy of this apex locator in the presence of NaOCl 2.5% and chlorhexidine 0.2% as the two common detergents which used in root canal treatment.

## Material and Methods

This study approved by ethical committee of Qazvin University of Medical Sciences.

fThe present study was an experimental laboratory study which performed on the extracted single root premolar tooth. Thirty extracted premolar tooth which had the criteria of the experiment (no root treatment, complete apex without fracture, lack of severe caries or complete obstruction) were collected. After rinsing, the tooth were placed in the NaOCl 5% solution for 24 hr. and then washed and stored in the 10% buffered formalin.

All decays were removed and occlusal surface was smooth to provide an exact and repeatable reference point. Then, access hole was prepared using fissure bur (Tess Kavan, Iran). Tooth were coded as 1 to 30 and then the coronal portion of canals was opened by Gates Glidden drills number 1 and 2 (Densply Co.).

K file number 10 (Mani Co., Japan) was inserted in the canal. When the file tip was seen from the apical foramen as tip to tip under the stereomicroscope with ×10 magnification, rubber-stop of the file was matched with occlusal reference point. Then, file was exited and length was measured using caliper. Afterward, 0.5 mm was diminished from this length and the value was recorded as the actual length of the canal.

Thereupon, 0.9% normal saline was used as electrolyte for electronic measurement by Root zx. Specific container was filled with this electrolyte and tooth were placed in the situation that the apical half of them were within normal saline.

Canals were being dried and then the first irrigant chlorhexidine 0.2% (Sahba Co, Iran) was used. One electrode was inserted in the saline container and another electrode was attached to the K file number 20 which inserted in the canal. Apparatus was run according to the manufacturer manual and the length to the apical constriction was measured triplicate and the mean of three measurement was considered as the final value. After replacing of irrigant and washing the canal by normal saline and drying, all of the above mentioned steps were repeated for NaOCl 2.5% which diluted from NaOCl 5% (Golrang Co., Iran). It must be noted that during all of measurements, the additional solutions were removed from the access cavity using cotton.

## Results

Mean difference between actual length and measured length by Root zx in the presence of chlorohexidine 0.2% was -0.055±0.498 mm while this mean difference in the presence of NaOCl 2.5% was 0.017±0.85 mm and showed no significant difference using Mann-Whitney U test (*P*=0.655,  Table 1). Positive values mean that file is shorter than apical foramen and negative values mean that file is outer the apical foramen. Zero value indicates that measured length by apex locator is equal to actual length. In this study using Root zx apex locator in the presence of chlorohexidine 0.2%, electronic measured length in 9 roots (30%) was equal to actual length. The electronic measured length in one root (3.3%) was 1 mm shorter, in 3 roots (10%) was between 0.5-1 mm shorter and in 3 roots (10%) was between 0.5-1 mm longer than actual length. In the presence of NaOCl 2.5%, the electronic measured length in one root (3.3%) was 1 mm shorter, in 9 roots (30%) was equal to and in 2 roots (6.7%) was between 0.5-1 mm longer than actual length ( Table 2).

**Table 1:**
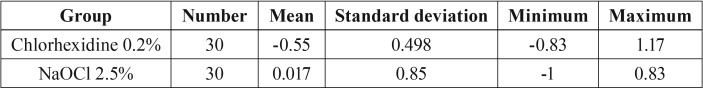
Mean of apical neck to file head distance in mm using Root zx apex locator in the presence of each solution.

**Table 2:**
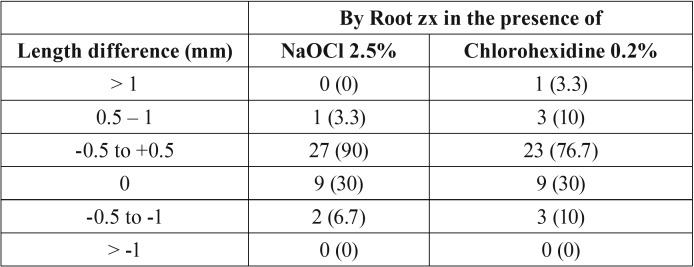
Distribution of difference of actual length and electronic measured length by Root zx apex locator (n (%)) in the presence of NaOCl 2.5% and chlorohexidine 0.2%.

Based on findings, accuracy power of Root zx apex locator for evaluation of root length with accepting of 0.5 and 1 mm errors in the presence of chlorohexidine 0.2 % was 76.7% and 96.7%, respectively. The accuracy of this system in the presence of NaOCl 2.5% with accepting of 0.5 and 1 mm errors in the presence of chlorohexidine 0.2 % was 90% and 100%, respectively ( Table 2).

There was no significant statistical difference between electronic measured and actual lengths in two different solutions (*P*=0.223).

## Discussion

Exact measurement of working length is one of the most important steps in the root treatment can be lead to successful treatment. There are several studies about the comparison of accuracy of measurement of working length by digital and conventional apparatuses (6-9). The first generation of apex locators worked based on resistance and the second generation of apex locators worked based on impedance (10). The most problem of these apex locators is the low accuracy in the presence of liquids and pulp tissue and also need to calibration before use. The third generation of apex locators are worked based on frequency and don’t need calibration. Their microprocessors calculated the impedance ration and therefore have high accuracy (11).

Root zx is a third generation apex locator which measured impedance in tow frequencies of 0.4 and 8 KHz and then calculated the impedance ratio and locate the file place in the canal (11). Root zx don’t need calibration and even can be used in the presence of strong ions in the canal. Saline environment which used in this study is one of the simplest method for *in vitro* measurement of the accuracy of apex locator and were used in different studies and confirmed its applicability (12,13). On the other hand, use of irrigant and chelating agent is one the most important parts of endodontic treatments (3). In the present study, the effects of two common irrigants included NaOCl 2.5% and chlorohexidine 0.2% on the accuracy of Root zx apex locator were evaluated.

In the study of Pagavia and colleagues on the accuracy of Root zx apex locator, firstly the working length of 29 teeth with live pulp which not prepared was measured by Root zx. Since the extraction of teeth with fixation of files in the canals, the working length was measured under electron microscopy. Accuracy in the group of teeth with apical foramen along the axis and root, was 100% with accepting of 0.5 mm error, and was 82.75% in the group of teeth with lateral apical foramen (14). In another study, Jenkins evaluated the accuracy of Root zx apex locator in the presence of lidocaine 2% contained 1:100,000 epinephrine, Rc-prep NaOCl 5.25%, EDTA, H2O2 3%, and peridex in 30 extracted teeth by Donnelly method. He found that all of the irrigants had no significant effects on the accuracy of this system and by accepting 0.31 error, no significant differences were existed between electronic measured and actual length in the presence of different irrigants (15). In the study conducted by Meares, by accepting of 0.5 mm error and using of 40 molar, premolar and incisor tooth with complete apex and 0.9% saline as experimental environment, the accuracy of Root zx apex locator in the presence of NaOCl 2.125%, 5.25% and absence of NaOCl were 83%, 85% and 81%, respectively (16). Moreover, Goldberg *et al.* investigated the accuracy of Root zx in the measurement of canal length of human teeth with an irregular cavity defect created at the apex of each tooth simulating an apical root resorption using saline as experimental environment. They found that in the presence of NaOCl 2.5% in the canals and accepting of 0.5 and 1 mm errors, the accuracy of system was 67.2% and 94%, respectively (17). On the other hand, it has been reported by Fouad that increase of the diameter of apical foramen can decrease the accuracy of Endex apparatus as another third generation apex locator (18).

Uzun et al. evaluated the effects of gutta-percha solvents which used in re-treatment on the accuracy of mini Root zx apex locator. They measured the actual length of 65 extracted maxillary incisor with mini Root zx apex locator. Then, teeth were worked by ProTaper system to actual working length. Twenty teeth were filled with gutta-percha and a resin-based sealer (Group A), 20 teeth with gutta-percha and a zinc oxide/eugenol-based sealer (Group B), and 16 roots were used as the control group (Group C) without filling. Then, removal of the root filling was done and re-preparation processes were performed using the ProTaper system. Guttasolv and Resosolv solvents in the group A and Guttasolv and Endosolv E solvents in the group B were used. After removing of the root fillings, the working length was measured again by Root zx. Their data showed that significant difference was existed between actual and electronic measured working length of A2 (Resosolv) and B2 (Endosolv) and electronic measured length was lower than actual length in them. Also, by accepting of 0.5 error, the accuracy of system in Resosolv group was clearly lower than other groups (19).

Berenice and collaborators compared the accuracy of two apex locators, Raypex 6 and mini Root zx. They selected mesial and buccal canal of 40 maxillary and mandibular molar and measured the canal length to apex by two apex locators (red line of system). Then, files were rewind to the green line in Root zx and yellow line in Raypex 6 (0.5 mm distance) and fixed and RVG radiography was performed. After that, one third of apical was shaved until exposure of the file and was measured at ×16 magnification. The average length from the tip of the file to the apical foramen was 0.695 mm using Root zx and 0.543 mm using Raypex 6 and had no statistical significant difference (20).

In an *in vitro* evaluation of the accuracy of Root zx apex locators (included Dentaport zx, Root zx Solfy ZX, TriAutozx) by Duh, it has been concluded that these apparatuses had high accuracy in determination of the place of the apical constriction and the maximum distance of file head to apical constriction was 0.1-0.19 mm. Totally, the accuracy of these sets in determination of apical constriction was 90.48-97.62% and no significant differences were existed between them (21). Finally, in the study by Kim and colleagues on the evaluation of working length by Root zx alone or in combination to radiography, it has been found that by accepting 0.5 mm error, Root zx could identify the place of apical constriction in 84% of sample but when combined with radiography, this level increased to 96%. However, this difference was not statistically significant (22). Overall *in vivo* accuracy of Root zx apex locator by accepting of 0.5 mm error were reported as 82.3% to 96.2% (3).

## Conclusions

The present study showed that Root zx apex locator in the presence of chlorohexidine 0.2% by accepting of 0.5 and 1 mm errors could identify the place of apical constriction with accuracy of 76.7% and 96.7%, respectively. In addition, in the presence of NaOCl 2.5% by accepting of 0.5 and 1 mm errors the place of apical constriction was identified accurately in 90% and 100% cases, respectively.
